# Support Services for Survivors of Ebola Virus Disease — Sierra Leone, 2014

**Published:** 2014-12-19

**Authors:** Seung Hee Lee-Kwan, Nickolas DeLuca, Monica Adams, Matthew Dalling, Elizabeth Drevlow, Gladys Gassama, Tina Davies

**Affiliations:** 1 Epidemic Intelligence Service, CDC; 2 Division of Nutrition, Physical Activity, and Obesity, National Center for Chronic Disease Prevention and Health Promotion, CDC; 3 Division of HIV/AIDS Prevention, National Center for HIV/AIDS, Viral Hepatitis, STD, and TB Prevention, CDC; 4 UNICEF, Sierra Leone; 5 GOAL, Sierra Leone; 6 Kenema Government Hospital, Kenema, Sierra Leone; 7 Sierra Leone Ministry of Social Welfare, Gender, and Children’s Affairs

As of December 6, 2014, Sierra Leone reported 6,317 laboratory-confirmed cases of Ebola virus disease (Ebola), the highest number of reported cases in the current West Africa epidemic (1). The Sierra Leone Ministry of Health and Sanitation reported that as of December 6, 2014, there were 1,181 persons who had survived and were discharged (2). Survivors from previous Ebola outbreaks have reported major barriers to resuming normal lives after release from treatment, such as emotional distress, health issues, loss of possessions, and difficulty regaining their livelihoods (3,4). In August 2014, a knowledge, attitude, and practice survey regarding the Ebola outbreak in Sierra Leone, administered by a consortium of partners that included the Ministry of Health and Sanitation, UNICEF, CDC, and a local nongovernmental organization, Focus 1000, found that 96% of the general population respondents reported some discriminatory attitude towards persons with suspected or known Ebola (5). Access to increased psychosocial support, provision of goods, and family and community reunification programs might reduce these barriers (3,6). Survivors also have unique potential to contribute to the Ebola response, particularly because survivors might have some immunity to the same virus strain (7). In previous outbreaks, survivors served as burial team members, contact tracers, and community educators promoting messages that seeking treatment improves the chances for survival and that persons who survived Ebola can help their communities (4). As caregivers in Ebola treatment units, survivors have encouraged patients to stay hydrated and eat and inspired them to believe that they, too, can survive (4,8). Survivors regaining livelihood through participation in the response might offset the stigma associated with Ebola (9).

The Sierra Leone Ebola Emergency Operations Psychosocial Consortium, which consists of members of the Sierra Leone government, nongovernmental organizations, and donor agencies, assessed survivors’ health, psychosocial, and financial needs, and their interest in supporting the Ebola response. In October 2014, the consortium assessed survivor needs in three districts (Bo, Kenema, and Bombali). Methods included 1) convening a National Survivor Conference in the Kenema District, where they conducted five focus groups with 36 survivors, 2) conducting in-depth interviews with 12 survivors, 3) conducting five additional district-specific focus groups with a total of 51 survivors, and 4) observing six survivor wellness center counseling sessions. The focus group discussions and in-depth interviews included assessing experiences as a survivor, support needed, support received when discharged from a medical facility, what they would tell other survivors of Ebola, what makes survivors feel special about having survived Ebola, and specific jobs or tasks survivors could perform. Data from summary findings from each of the 10 focus groups, 12 in-depth interviews, and six direct observation field notes were reviewed and coded to identify emerging themes.

Common themes that emerged were immediate and long-term concerns about physical and mental health, stigma, psychosocial issues, reintegration needs, and financial needs. Survivors reported health problems; the most common symptoms reported were blurred or partial loss of vision, dizziness, headache, sleeplessness, and myalgia. Survivors who reported physical health issues after recovery expressed interest in receiving medical attention specific to reported post-Ebola health issues. Survivors also raised concerns regarding psychosocial issues (e.g., stigma and shame that prevents reintegration into their community, as well as survivor guilt) and financial burden. Many Ebola survivors had most of their belongings burnt or taken away as part of infection control, including their clothing and household goods. Many reported being shunned by the community and had difficulty accessing shops to purchase replacement goods. Survivors emphasized the critical need for comprehensive discharge counseling and the provision of a packet of materials, including clothing and cash for transportation, as well as facilitation of reentry into the community by professional psychosocial support counselors.

Survivors showed great interest in contributing to the Ebola response through activities like sharing their stories directly with their community, with Ebola patients currently receiving care, or with a larger audience through radio and other broadcast media. They also expressed interest in participating in Ebola care and treatment support and direct care, and providing moral support to other Ebola patients to give them hope. Many indicated that supporting themselves with this work would help restore their own dignity.

Upon completion of the assessment, findings were shared with select district-level Emergency Operations Center staff and partners involved in the response to improve and coordinate the survivor services. To address commonly reported sequelae of Ebola, the nongovernmental organization Sight Savers (http://www.sightsavers.org) is piloting the provision of free eye examinations and treatment for survivors with vision problems in select districts. The services will be rolled out nationally in the coming months. The Sierra Leone Ebola Emergency Operations Psychosocial Consortium also is coordinating partners and districts to improve the initial and ongoing psychosocial support for survivors. A counselor-client flipbook that contains a series of pictures with information to help change health behaviors is in development and will serve as an aid for counselors to ensure consistent and comprehensive discharge planning and counseling for all survivors throughout Sierra Leone. Likewise, a comprehensive survivor packet has been designed to ensure the consistent provision of resources to survivors upon discharge. The packet includes a mattress, bed sheets, a blanket, a towel, a pillow, a water bucket, a cell phone, utensils, a cooking pot, laundry soap, bar soap, a toothbrush and toothpaste, a mosquito net, a set of clean clothes and under garments, plastic sandals, food, cash, condoms, and multivitamins.

To assist with survivor reintegration in the community, the consortium recommends that counselors accompany survivors when returning to their home village after discharge to facilitate reunification and reintegration of survivors into their communities. The reintegration process also includes trained counselors speaking with local traditional authorities and other community members about the survivor’s status, the importance of survivor acceptance, and ways the community can support the survivor. In addition, stigma mitigation educational materials targeting the community have been developed and implemented, including 1) various media channels highlighting survivor stories and testimonials, 2) training of district nongovernmental organizations to address stigma, and 3) training of trainers of psychosocial support counselors to use interpersonal communication materials during community engagement activities.

Finally, national and local health officials have started considering the roles Ebola survivors can serve as part of Ebola outbreak response. The Sierra Leone Ebola Emergency Operations Psychosocial Consortium is coordinating the distribution of comprehensive discharge counseling, reintegration services, and packet distribution and establishing survivor support centers and services at the district level. Further monitoring and guidance from international partners regarding best practices will inform next steps for supporting Ebola survivors, potentially integrating them further into response activities.

## References

[1] CDC 2014 Ebola outbreak in West Africa—case counts.

[2] National Ebola Response Centre (NERC) (2014). Ebola outbreak updates.

[3] De Roo AD, Ado B, Rose B, Guimard Y, Fonck K, Colebunders R (1998). Survey among survivors of the 1995 Ebola epidemic in Kikwit, Demographic Republic of Congo: their feelings and experiences. Trop Med Int Health.

[4] Hewlett BS, Hewlett BL (2008). Ebola, culture, and politics: the anthropology of an emerging disease.

[5] Focus 1000 Study on public knowledge, attitudes, and practices related to Ebola virus disease prevention and medical care in Sierra Leone.

[6] Reaves EJ, Mabande LG, Thoroughman DA, Arwady A, Montgomery JM (2014). Control of Ebola virus disease—Firestone District, Liberia, 2014. MMWR Morb Mortal Wkly Rep.

[7] Wong G, Kobinger GP, Qiu X (2014). Characterization of host immune responses in Ebola virus infections. Expert Rev Clin Immunol.

[8] Daily Nation (Kenya) I survived Ebola to help others fight the disease.

[9] Hewlett BS, Amola RP (2003). Cultural contexts of Ebola in Northern Uganda. Emerg Infect Dis.

